# Therapeutics

**Published:** 1875-11

**Authors:** 


### VI. Therapeutics.

1. *Sea Sickness—Its Pathology and Treatment with Amyl Nitrite.* Crochley Clapham, L.R.C.P.L., Ph.D., etc. (*Lancet.*)

The writer has had the opportunity of making a post-mortem examination in a case of this malady—the only one we believe on record. The subject was a Chinaman, who was *instantly* killed while vomiting, by a falling piece of iron. Autopsy four hours after death. "All the organs were healthy, excepting the spinal cord, the vessels of which were literally gorged with blood throughout its entire length." J. Crichton Browne, found a similar congestion in an epileptic patient, who died in the "status." He used amyl nitrite in similar cases advantageously. Dr. Clapham accordingly used amyl nitrite in *nausea marina* with astonishing success. Out of 124 cases, 121 were permanently relieved after one inhalation, (of three to five drops), and the other three were relieved permanently after a second or third inhalation.

2. *Quinine as a Gargle in Diphtheritic, Scarlatinal and Other Forms of Sore Throat.* DAVID J. BRAKENRIDGE, M.D., F.R.C.P.E. (*Practitioner*, August, 1875.)

The following points are considered as established with regard to the action of quinine on the white blood corpuscles and blood vessels.

"1st. Quinine is a protoplasm poison, and limits the number and movements of the white blood corpuscles and pus cells.

"2d. It prevents the migration of the blood corpuscles into the tissues of the membranous and parenchymatous organs exposed to the air, both when it is given subcutaneously and when it is applied directly to the part.

"3d. It restrains the dilatation of the blood vessels.

"4th. It is antiseptic, and exerts a paralyzing, or, in larger doses, a destructive influence on microzymes."

Basing his practice on the above ideas, the author has used quinine gargles in various sore throats. "In the slighter forms of diphtheritic sore throat it answers admirably, preventing the extension of the disease, and promoting the separation of the membranous exudation." As a rule, he used the strength of two grains to the ounce of water, with five drops of dilute sulphuric acid. "Sometimes I have been able to increase the strength; sometimes I have been compelled to diminish it. When well tolerated, the stronger it is the better."

"Non-syphilitic ulcers of the throat, under this treatment, at once assume a healthier aspect and heal rapidly." "Its effects in scarlatinal sore throat are very marked, the pultaceous secretion being checked, and the inflammatory swelling diminished." In severe cases, he recommends gargling every hour.

3. *Gelseminum Sempervirens in Facial Neuralgia.* C. H. NEWCOMB, A.M., M.D., Mechanicsburg, Ohio. (*Cin. Lancet and Observer*, September, 1875.)

This drug is vaunted very highly in the above disease, and several cases detailed. The dose used varies from five to ten drops of the fluid extract every three or four hours. The result always was relief. He thinks six drops of the fluid extract every four hours sufficient. This drug, the writer considers, has as certain an influence over the nerves of the head and face, as ergot has over the uterus. To physicians in malarial districts, he would recommend gelseminum in the facial neuralgias that resist iron, quinia, strychnia and arsenic. Its cheapness is a secondary consideration, and of considerable importance to those who have to supply their own drugs.

4. *Gelseminum in Odontalgia.* JAMES SAWYER, M.D., Land. M.R.C.P. (*Practitioner*, August, 1875.)

"I have rarely found gelseminum fail to give decided and lasting relief in cases of neuralgic pains in the face and jaws, associated with carious teeth. I have usually given fifteen minims of the tincture every six hours."

5. *Arrest of Cardiac Palpitations.* ANON. (*Med. Times and Gazette*, August 28, 1875.)

A correspondent in the *Union Médicale*, August 21, calls attention to the fact that palpitations, when not dependent upon organic disease, may be almost immediately arrested by bending the head downwards and allowing the arms to hang pendant. The effect is even still more rapidly produced by holding the breath for a few seconds, while the body is in this bent position.

